# A Brief Report from the Clinics of Berlin

**Published:** 1854-11

**Authors:** 


### *A brief report from the Clinics of Berlin. Clinic of* Dr. Graefe.

(Translated for the Examiner by Dr. Theo. C. Hilgard.)

### *Wednesday, Nov.* 16*th*, 1853. *Operation.*

*Two cases of Extraction of Cataract.*—In common senile cataract, Graefe considers extraction the most superior method of operation; according to his views, dislocation is indicated only in exceptional cases, and he gives as a reason, extensive statistical experiences on the results of operations. The danger incurred by reclination consists chiefly in those protracted internal inflammations, which, caused by the irritation produced on the internal membranes by the reclined lens, so frequently impair or destroy altogether the eye-sight previously obtained. A reclined lens acts in the same manner as a foreign body in a higher or lesser degree; is not within the reach of the operator, but governed by uncontrollable contingencies. Thus after the most successful operations with the needle, we frequently see melancholy results. Nor are many authors less mistaken in the time required for the absorption of reclined lenses; though this is extremely variable, it has been determined that a semi-indurated lens is never absorbed in less than nine months; in many cases years are required, and that in many others it does not take place at all, the alienated lens being retained within the eye, enclosed in an exudation. In contra-distinction to this in extraction of the lens, the ingression takes place but once, and is directly dependent on the operation. Its results are free from subsequent and insidious diseases, and are more under the control of the operator. On the other hand, the technical part thereof is much more difficult, and requires a profound and minute study. Dr. Graefe prefers the *upward* corneal section, on account of the better position of the lobe and the homogenous compressive dressing that nature supplies in the upper eyelid; various other advantages cited of this method, are either contained in the above or do not altogether hold good. The latter applies, *e. g.*, to the argument, that scars of the cornea would be less inconvenient in the upper than in the lower part; but if any scar at all is left, it must be caused by a prolapse of the iris, combined with a distortion of the pupil; the latter being *cæteris paribus* more inconvenient if it be an upward dislocation of the pupil, than if it be the reverse. If suppuration in the wound once ensues with an upward cut, it is more destructive by the subsidence of matter into the anterior chamber, which also renders it more difficult to save the eye for a coremorphose.* The two latter circumstances, then, would rather argue in favor of extraction downwards. Nevertheless, Graefe considers the advantages of the upper section to be decidedly greater.

Respecting the season, Graefe contradicts the prejudice so current among the public and many physicians, that summer time is best adapted to the operations for cataract; as the number of inflammatory diseases of the eyes in our climate, (Prussia,) is far more considerable in summer, so is the tendency to inflammation after operations. The hot months (July and August,) are to be avoided, and June, also. The first months of spring are more favorable, but present no advantages above autumn and winter, excepting that the patient may leave his room a few days earlier. The cases were then operated on.

*Saturday, Dec. 10th*, 1853.—After presenting the current clinical cases, Dr. Graefe proceeded to speak of the treatment of conjunctival granulations. In those slight elevations, occasioned in chronic catarrhs by erection of the normal vascular loops, and an accumulation of blastema around them, nitrate of silver forms the chief remedy. Generally speaking, the application of strong solutions, more rarely repeated, is preferable to the frequent application of weak ones. The lids are turned over, and a solution of gr. x. to unc. i. is energetically applied to the conjunctival surface. After a few seconds' action the solution is washed away with water, and the lids are replaced. If there be a strong mucous excretion, it is preferable not to wash away the application, but to leave it on the lid to produce a more energetic action. In cases of much secretion, also, the conjunctiva is less irritated by this agent, and the pain is less. If *blepharitis marginalis* and *angularis*, which so frequently accompany chronic conjunctivitis, be co-existent, the solution is also brushed over the excoriated cutaneous parts. After the

* Operation for artificial pupil.

application, cooling the eyes with cold water may be recommended in general, as long as the presence of pain makes it pleasant to the patient. The interval of repetition is very different in different cases; namely, after the pain caused by cauterization has subsided, a remission of the subjective, as well as objective, morbid phenomena is observable; whilst a relapse of the disease will supervene after a certain duration of the remission. This period must, however, not be waited for, if a prompt curative effect be desired, but the caustic solution must be reapplied during the time of remission; and, therefore, no absolute scheme of treatment can be given. In some cases, the remedy must be applied twice a day; whilst in others, a repetition, even after two days, has still a super-irritative action. To a total disregard or imperfect valuation of this fact it is owing, that some practitioners will have nothing at all to do with this mode of treatment, which Graefe, however, has tested by comparison in several hundred cases, where both eyes being equally affected, different curative trials were instituted on each eye, from which he has drawn the conviction that although not in all, yet in a decided majority of cases, the treatment above stated yields the quickest cures. In cases of little secretion and great irritability of the eyes, five grains are used for the solution, instead of ten. Only if the reddening of the mucous membrane, caused by the solution of nitrate of silver, exceeds the usual limits, Graefe uses solution of acetate of lead, on an average of one scruple to an ounce.

An acute and vivid injection of the conjunctiva of the globe of the eye by no means contraindicates the above mentioned treatment; only the diagnosis must be such as not to permit of doubt, that it actually is the conjunc. that forms the focus of the inflammation; and that it does not, as so frequently is the case, appear so, from being symptomatic of some morbid affection of other parts of eye. On the contrary, the energetic application of nitrate of silver forms in most cases of acute conjunctivitis, an abortive treatment that renders the application of all other remedies superfluous, and timely encounters the development of chronic and obstinate diseases of the eye. However, we only refer here to the diffuse, or so-called catarrhal conjunctivitis, whilst other forms, and in particular phlyctænular or pustular conjunct., does not, generally speaking, bear caustic applications; but, in general, the principle obtains that the more violent the conjunctival injection is, the stronger should be the caustic application; and we find also that a weak solution applied timidly generally increases the irritation in the acute stages, while strong ones quench all the inflammatory phenomena in a marvellously prompt manner. Not unfrequently, after twenty four hours, an eye, previously violently injected, will be found completely colorless.

These facts, however, present a perfect analogy with what occurs in inflammations of other mucous membranes, for instance of the urethra; but in no case can the action of caustics be studied so well as in the mucous membrane of the eye, whose entire surface is accessible to our view. Complications with other affections, for instance of the cornea, by no means however, absolutely exclude the said method; only it essentially depends on the exact seat, and the nature of the produced exudation, of which Graefe treats afterwards.

In the severer granulations of chronic blennorrhœa, nitrate of silver *in substance*, mixed with saltpetre, is preferable, as it permits us to localize and concentrate the effect to the parts most diseased. After the application of the caustics, one must always neutralize with salt water, which, after that, is washed out of the eye with water. If the injection and pain continue for a long time, scarifications after each cauterization, are to be employed. Graefe never applies the pure stick of nitrate of silver, as it affects the mucous membrane too much, and disposes it to scarry (cicatrized?) shrinking. Next to the nitrate of silver, a concentrated solution of *acetate of lead*, scr. ii. to unc. j., is most to be recommended, as it occasions but little pain; and therefore, is suitable for irritable eyes. The *sulphate of copper*, so extensively used now-a-days, is also a very valuable remedy. In many cases, an occasional change of remedies is to be recommended; for, indeed, the mucous membrane seems to become gradually blunted, as it were, to the beneficial action of each single means, sooner or later. We here observe, although less distinctly marked, something similar to what we observed in the mydriatic alkaloids; sulphate of daturina and sulphate of atropina, if well prepared, not only exert no irritating effect upon the normal eye, but they also form an invaluable part of the antiphlogistic curative apparatus in more profound ophthalmia; yet, after several weeks' application, the eye becomes so irritable towards them, that, as if saturated with them, it reacts after each drop of the diluted solution by a violent conjunctivitis; a species of saturation, which even in an apparently normal state of irritability of the eye, sometimes continues through months, if not removed by other topical remedies.

In the trachomatous granulations, the same agents are to be applied. The solid nitrate of silver must, however, be used with great care, if sound parts of the mucous membrane have begun to appear. In such cases, *sulphate of copper* or a *solution of nitrate of silver* deserve preference. Very good results are also obtained by concentrated solutions of *acetate of lead*; whilst neither the touching nor the powdering of the mucous membrane with them, can by any means be praised, notwithstanding the warm recommendations of them from Belgium.—*Extracted from Deutsche Klinik, No.* 48.

